# Effect of Two Seeding Rates on Nitrogen Yield and Nitrogen Fixation of Winter and Spring Faba Bean

**DOI:** 10.3390/plants12081711

**Published:** 2023-04-20

**Authors:** Reinhard W. Neugschwandtner, Alexander Bernhuber, Stefan Kammlander, Helmut Wagentristl, Agnieszka Klimek-Kopyra, Tomáš Lošák, Jaroslav Bernas, Lukas J. Koppensteiner, Kuanysh K. Zholamanov, Mohammad Ghorbani, Hans-Peter Kaul

**Affiliations:** 1 Institute of Agronomy, Department of Crop Sciences, University of Natural Resources and Life Sciences Vienna (BOKU), Konrad-Lorenz-Straße 24, 3430 Tulln, Austria; 2 Experimental Farm Groß-Enzersdorf, Department of Crop Sciences, University of Natural Resources and Life Sciences Vienna (BOKU), Schloßhoferstraße 31, 2301 Groß-Enzersdorf, Austria; 3 Institute of Crop Production, University of Agriculture of Cracow, Al. Mickiewicza 21, 31-120 Cracow, Poland; 4 Department of Environmentalistics and Natural Resources, Faculty of Regional Development and International Studies, Mendel University in Brno, Zemědělská 1, 61300 Brno, Czech Republic; 5 Department of Agroecosystems, Faculty of Agriculture and Technology, University of South Bohemia, Branišovská 1457, 37005 České Budějovice, Czech Republic; 6 Department of Land Resources and Cadastre, Faculty of Water, Land and Forest Resources, Kazakh National Agrarian University (KazNAU), Abai Avenue 8, Almaty 050010, Kazakhstan

**Keywords:** *Vicia faba*, extended difference method, nitrogen sparing, soil mineral nitrogen, nitrogen balance, nitrogen utilization

## Abstract

Faba bean (*Vicia faba* L. *minor*) is an important grain legume and is widely used as food and feed. It is traditionally used as a spring crop in Central European cropping systems. There is increasing interest in winter faba bean due to a higher yield potential, but limited knowledge of nitrogen (N) yields and nitrogen fixation (N_FIX_) exists. Therefore, the purpose of this study was to compare N concentrations, N yield of plant fractions, soil mineral N (SMN) and SMN sparing in the soil after harvest, N_FIX_ and N balance of two winter faba bean varieties (Diva and Hiverna) to those of a spring faba bean (Alexia) using two seeding rates (25 versus 50 germinable seeds m^−2^) in a two-year field experiment under Pannonian climate conditions in eastern Austria. The winter faba bean varieties had higher N yields and N_FIX_, not only due to higher biomass yields, but also due to higher N concentrations and a higher percentage of N derived from atmosphere in the biomass. Conversely, the soil mineral N after harvest was lower compared to the spring faba bean. All treatments had a negative N balance due to higher grain N yield than N_FIX_. Winter faba beans left higher amounts of biologically-fixed N in residues for the subsequent crop, whereas spring faba bean left more SMN. Winter faba bean varieties obtained good results with both seeding rates, whereas the grain yield and the grain N yield of Alexia tended to higher with the higher seeding rate.

## 1. Introduction

Faba bean is a protein-rich grain legume which is widely used as food and feed for monogastric animals and ruminants. It is well adapted to most climatic areas of Europe [1]. However, the yield variability, due to factors including heat and drought stress, impede the wide adoption of faba bean in European farming systems [2]. Flowers and young pods of faba bean can be damaged by environmental stresses such as frost, heat and drought. Therefore, adapting phenological development to avoid unfavourable environmental conditions is important for minimizing their detrimental effects [3]. Autumn-sowing of faba bean, which is widely carried out in France and the United Kingdom [4], could be an alternative to the common spring-sowing of faba bean in Central Europe to minimize negative heat and drought effects. Under Central European climate conditions, autumn-sown crops flower earlier, growth stages for yield formation last longer and yields are higher when compared to spring-sown crops, as shown for facultative wheat [5,6] and facultative triticale [7]. Also, autumn-sowing of winter forms of the traditional spring crops faba bean and pea have resulted in higher yields and higher nitrogen (N) uptake than spring-sowing [8,9,10,11].

Further, climate change is expected to result in higher temperatures all year round and a lower total annual amount of precipitation in Central Europe, but may also cause more precipitation in winter and early spring and less precipitation in summer. This will make the cultivation of spring crops much more challenging in Central Europe [12]. Fostering winter faba bean inclusion into cropping systems might, therefore, be an adaptation strategy for addressing challenges arising from climate change. In a simulation study for conditions in south-western France, a scenario with a decrease in rainfall and an increase in temperature estimated a strong yield decrease of spring pea and a lower decrease for winter pea, whereas the yield of winter faba bean remained unaffected [2].

Grain legumes strongly contribute to the diversification, sustainability and long-term productivity of agricultural systems, due to the biological nitrogen fixation (N_FIX_) of atmospheric N through symbiosis with N-fixing soil bacteria, from which they can satisfy the bulk of their N demand [13]. N_FIX_ plays a critical role in crop production, providing almost 20% of worldwide needed N for grain and oilseed crop production [14]. 

N_FIX_ can be increased by agronomic management, for example, by attaining higher yields through optimizing sowing dates and plant density [15]. For soybean, late sowing was reported to reduce the protein yield [16]. In Syria, chickpea had a considerably higher proportion of N derived from N_FIX_ with autumn-sowing compared to spring-sowing [17]. Increasing competition for soil mineral N at a higher plant density can result in an increased N_FIX_. This has been observed especially in cereal–legume intercrops, where the legume’s proportion of N derived from N_FIX_ compared to the corresponding sole crop has been shown to be increased [18] because the legume is forced to rely more strongly on N_FIX_, as the cereal in intercrops is more competitive for soil inorganic N [19]. Competition through higher plant density increased N_FIX_ also in sole faba bean crops, although the effect was not as high as in intercrops of faba bean with barley [20]. High plasticity to adapt N_FIX_ to different plant densities has been shown for soybean, with a higher nodule number and higher total nodule dry weight per plant, with a lower plant density [21].

Plant density is one of several agronomical practices that need to be addressed for the improvement of faba bean production in European cropping systems [22]. Optimum seeding rates are different for winter and spring faba bean. In a 3-year experiment in Poland, spring faba bean grown with 75 compared to 45 seeds m^−2^ showed a higher seed yield in 1 and a higher residue yield in 2 years [23]. However, winter faba bean grown in Spain increased the seed yield from 10 to 16 plants m^−2^, but not further than 21 plants m^−2^ [24]. For the present study, yields and formation of yield components of winter and spring faba bean grown in Central Europe, as affected by seeding rate (25 versus 50 germinable seeds m^−2^), have already been reported [11]: the grain yield (Y_GRAIN_) of winter faba bean varieties were over all seeding ratios and years 1.71-fold (Diva) or 1.33-fold (Hiverna) higher compared to the spring faba bean Alexia. Winter faba bean varieties had further higher values for above-ground dry matter yield, stem density, number of stems plant^−1^, thousand kernel weight (TKW) and grain yield pod^−1^. Seeding rates (25 versus 50 germinable seeds m^−2^) did not affect the Y_GRAIN_ of Diva, but the Y_GRAIN_ of Hiverna was higher at the lower seeding rate, while that of Alexia tended to be higher at the higher rate. The higher seeding rate resulted in a higher plant density and stem density but reduced the number of stems plant^−1^ (of winter faba bean varieties) and the number of pods plant^−1^.

Limited knowledge is available for N uptake and N_FIX_ of winter faba bean in general and particularly of optimal seeding rates under Central European growing conditions. Therefore, the aim of this study was to compare winter and spring faba bean in eastern Austria in order to gain knowledge on N uptake and N_FIX_ under contrasting sowing dates as affected by seeding rates.

## 2. Materials and Methods

### 2.1. Environmental Conditions

The experiment was performed at the experimental farm Groß-Enzersdorf of the University of Natural Resources and Life Sciences Vienna (BOKU) in Raasdorf (48°14′ N, 16°35′ E; 154 m above sea level) on a chernozem soil of alluvial origin which is rich in calcareous sediments (pH_CaCl2_ = 7.6, silty loam, 2.2–2.3% organic matter). Soil mineral nitrate (NO_3_-N) in 0–0.9 m depth at sowing was at 21.7 g m^−2^ (17 October 2013), 13.0 g m^−2^ (13 October 2014), 18.4 g m^−2^ (4 March 2014) and 11.2 g m^−2^ (16 March 2015).

The mean annual temperature is 10.7 °C and the mean annual precipitation is 543 mm (1983–2012). The temperature in the vegetation period of winter crops (October to July) in both years was above the long-term average and the precipitation was, in the first experimental year, above and, in the second, below the long-term average. For monthly means, see Neugschwandtner et al. [8].

### 2.2. Experimental Setup and Treatments

Winter faba beans varieties Diva and Hiverna were sown in October and the spring faba bean variety Alexia in March. Seeding rates were 25 or 50 germinable seeds m^−2^. Winter wheat Xenos was grown as a reference crop for N_FIX_, with 300 germinable seeds m^−2^. Crops were harvested from end of June to mid-July. No fertilization was performed. The experiment was performed in the years 2013/14 and 2014/15. Detailed information on varieties, weed and pest control, seeding and harvest dates is given in Neugschwandtner et al. [8].

### 2.3. Nitrogen Determination and Calculations

The N concentrations of grain (NC_GRAIN_), pod walls (NC_PW_) and stems and leaves (NC_SL_) were determined as an average of duplicate samples of about 50 mg each, via the Dumas combustion method [25] using an elemental analyzer (vario MACRO cube CNS; Elementar Analysensysteme GmbH, Germany). The N yields (NY) were calculated by multiplying biomass yields with N concentrations. The N harvest index (NHI) was calculated as the percentage share of the grain N yield (NY_GRAIN_) on the above-ground dry matter NY. The N utilization efficiency for the production of AGDM (NUtE_AGDM_) or grain (NUtE_GRAIN_) was calculated according to Sinebo et al. [26] as follows:

(1) $${\mathrm{NUtE}}_{\mathrm{AGDM}}\;\left({g\;g}^{-1}\right)=\frac{Y_{\mathrm{AGDM}}}{{\mathrm{NY}}_{\mathrm{AGDM}}}$$

(2) $${\mathrm{NUtE}}_{\mathrm{GRAIN}}\;\left({g\;g}^{-1}\right)=\frac{Y_{\mathrm{GRAIN}}}{{\mathrm{NY}}_{\mathrm{AGDM}}}$$

where Y_AGDM_ or YG_RAIN_ are the yields of AGDM or grain and NY_AGDM_ is the N yield of the above-ground dry matter (AGDM).

Soil sampling after harvest was performed to a depth of 0.9 m with soil probes (Puerckhauer type, core diameter: 30 mm). Samples were further divided into three layers: 0–0.3, 0.3–0.6 and 0.6–0.9 m. Soil samples were extracted with 0.0125 M CaCl_2_ (*w*/*v* = 1:4) for 1 h using an overhead shaker [27]. The residual soil mineral nitrogen (SMN, as nitrate-nitrogen: NO_3_-N) was determined photometrically (FIASTAR 5000, FOSS GmbH, Hamburg, Germany).

The symbiotically fixed N per unit area (N_FIX_) at harvest was calculated by comparing the differences of the NY_AGDM_ (∆ NY_AGDM_) and the SMN depletion (SMN sparing) in the soil (0–0.9 m) between the legume and non-legume reference crop (winter wheat), using the extended difference method [28] as follows:

N_FIX_ (g m^−2^) = (NY_AGDM-Leg_ − NY_AGDM-Ref_) + (SMN_Leg_ − SMN_Ref_) (3)

where NY_AGDM-Leg_ and NY_AGDM-Ref_ are the quantities of N in the above-ground dry matter and SMN_Leg_ and SMN_Ref_ are the amounts of SMN of the legume and the reference crop. All values are in g m^−2^. The lower removal of SMN by the legume compared to a cereal during crop growth is termed as ‘nitrogen sparing’ [29]. The NY_AGDM-Ref_ was 11.7 (2013/14) or 13.0 (2014/15) g m^−2^ and the SMN_Ref_ was 3.33 (2013/14) or 0.14 (2014/15) g m^−2^. 

The percentage of the NY_AGDM_ derived from atmospheric fixation (Ndfa) in the AGDM was calculated according to Urbatzka et al. [30] as follows:

Ndfa (%) = NY_AGDM_/N_FIX_ × 100 (4)

The N_FIX_ per unit of AGDM (N_FIX-AGDM_) of faba bean at harvest was calculated as follows:

N_FIX-AGDM_ (mg g^−1^) = N_FIX_ (g m^−2^)/AGDM (g m^−2^) × 1000 (5)

The N balance was calculated as the difference between N_FIX_ and N removal of the grain according to Wichmann et al. [31] as follows:

N balance (g m^−2^) = N_FIX_ (g m^−2^) − NY_GRAIN_ (g m^−2^) (6)

### 2.4. Statistics

The experimental setup was a randomized complete block design with 4 replications and an individual plot area of 15 m^2^ (10 × 1.5 m). Analysis of variance using a nested design with replications nested within years was performed with SAS version 9.2. Means were separated by least significant differences (LSD) when the F-test indicated factorial effects at the significance level of *p* < 0.05.

## 3. Results

### 3.1. Biomass Yields

The mean biomass yields over all varieties, seeding rates and years were as follows: Y_GRAIN_: 272 g m^−2^, yield of pod walls (Y_PW_): 59 g m^−2^, yield of stems and leaves (Y_SL_): 377 g m^−2^.

The Y_GRAIN_ ranked among varieties at different seeding rates as follows: 25—Diva > Hiverna > Alexia; 50—Diva ≥ Hiverna ≥ Alexia. The Y_GRAIN_ of Diva and Hiverna were 1.68-fold or 1.32-fold higher compared to Alexia (over all seeding rates and years). Diva had a higher Y_GRAIN_ at 25 when compared with 50 seeds m^−2^; Alexia tended to be higher with 50 than with 25 seeds m^−2^, whereas Hiverna was not affected by seeding rate. The Y_GRAIN_ did not differ between the years (Table 1). 

The Y_PW_ was ranked as follows: variety—Diva > Hiverna, Alexia; year—2014/15 > 2013/14, with no effect of seeding rate (Table 1). 

The Y_SL_ was ranked among varieties as follows: Hiverna > Diva > Alexia, with higher values for Diva in 2013/14 than in 2014/15 and no difference between years for Hiverna and Alexia. Y_SL_ was higher at 50 than 25 seeds m^−2^ (Table 1 and Table 4).

### 3.2. Nitrogen Concentrations and Nitrogen Yields

The mean values for NC and NY over all varieties, seeding rates and years were for NC_GRAIN_: 4.64%, NC_PW_: 1.44%, NC_SL_: 0.91%, NY_AGDM_: 17.1 g m^−2^, NY_GRAIN_: 12.7 g m^−2^, N yield of pod walls (NY_PW_): 0.83 g m^−2^, N yield of stems and leaves (NY_SL_): 3.59 g m^−2^, N yield of total residues (NY_TR_): 4.42 g m^−2^ and NHI: 73.8%.

The NC_GRAIN_ was ranked among varieties as follows: 2013/14—Diva > Alexia, Hiverna; 2014/15—Diva > Hiverna > Alexia, with a higher concentration for Diva in 2014/15 than in 2013/14 and no difference between years for Hiverna and Alexia. The NC_GRAIN_ of Diva and Hiverna were 1.08-fold or 1.04-fold higher compared to Alexia (over all seeding rates and years) (Table 1 and Table 4). 

The NC_PW_ of Diva was higher than of that of the other varieties in 2014/15 than in 2013/14. No difference between varieties occurred in 2013/14 and Hiverna and Alexia had similar concentrations in both years (Table 1 and Table 4). 

The NC_SL_ was ranked as follows: variety—Hiverna ≥ Alexia ≥ Diva; year—2013/14 > 2014/15. The NC_SL_ of Diva tended to be considerably lower than that of Hiverna and Diva in 2013/14, whereas in 2014/15, differences between varieties were smaller (*p* = 0.063, n. s.) (Table 1 and Table 4). 

Seeding rates did not affect the NC_GRAIN_, NC_PW_ and NC_SL_ (Table 2).

The NY_AGDM_ and the NY_GRAIN_ were ranked among varieties as follows: NY_AGDM_—Diva, Hiverna > Alexia; NY_GRAIN_—Diva > Hiverna > Alexia. The NY_AGDM_ of Diva and Hiverna were 1.73-fold or 1.58-fold higher compared to Alexia and the NY_GRAIN_ were 1.80-fold or 1.37-fold higher (over all seeding rates and years). No significant differences occurred between seeding rates and years, but the NY_GRAIN_ showed a tendency to be higher in 2014/15 than in 2013/14 (*p* = 0.051, n. s.). For Alexia, both the NY_AGDM_ (*p* = 0.066, n. s.) and the NY_GRAIN_ (*p* = 0.059, n. s.) also tended to be higher at 50 than at 25 seeds m^−2^, with lower differences between seeding rates for Diva and Hiverna (Table 1 and Table 3). 

The NY_PW_ was ranked as follows: variety—Diva > Hiverna, Alexia; year—2014/15 > 2013/14. No differences occurred between seeding rates (Table 2). The Y_SL_ and NY_TR_ were ranked as follows: variety—Hiverna > Diva > Alexia; seeding rate—50 > 25; year—2013/14 > 2014/15. The NY_TR_ of Diva and Hiverna were 1.49-fold or 2.27-fold higher compared to Alexia (over all seeding rates and years) (Table 2).

**Table 1:** Yields, N concentrations and N yields of faba bean as affected by variety, seeding rate and year.

	Y_GRAIN_	Y_PW_	Y_SL_	NC_GRAIN_	NC_PW_	NC_SL_	NY_AGDM_	NY_GRAIN_	NY_PW_	NC_SL_	N_TR_	NHI
	g m^−2^			%			g m^−2^					%
**Variety (V)**												
Diva	344 ^a^	79 ^a^	361 ^b^	4.81 ^a^	1.39 ^b^	0.81 ^b^	20.6 ^a^	16.5 ^a^	1.08 ^a^	3.06 ^b^	4.14 ^b^	79.2 ^a^
Hiverna	269 ^b^	53 ^b^	547 ^a^	4.64 ^b^	1.39 ^b^	1.01 ^a^	18.8 ^a^	12.5 ^b^	0.73 ^b^	5.59 ^a^	6.33 ^a^	65.7 ^b^
Alexia	205 ^c^	44 ^b^	224 ^c^	4.47 ^c^	1.53 ^a^	0.91 ^ab^	11.9 ^b^	9.1 ^c^	0.67 ^b^	2.11 ^c^	2.78 ^c^	79.4 ^a^
**Seeding rate (S) (m^−2^)**												
25	274	59.8	350 ^b^	4.63	1.42	0.88	16.8	12.8	0.83	3.19 ^b^	4.02 ^b^	75.6
50	271	58.0	405 ^a^	4.64	1.46	0.94	17.4	12.6	0.83	3.98 ^a^	4.81 ^a^	72.0
**Year (Yr)**												
2013/14	262	51 ^b^	414	4.56 ^b^	1.38 ^b^	1.15 ^a^	14.4	11.9	0.70 ^b^	4.80 ^a^	5.50 ^a^	67.9 ^b^
2014/15	283	66 ^a^	340	4.72 ^a^	1.49 ^a*^	0.67 ^b^	16.8	13.5	0.96 ^a^	2.37 ^b^	3.34 ^b^	79.7 ^a^
**ANOVA**												
V	***	***	***	***	**	*	***	***	***	***	***	***
S			***							*	*	
Yr		**	***	***	*	***			***	***	***	***
V × S	*											
V × Yr			***	***	*							*

Y = yield, NC = nitrogen concentration, NY = nitrogen yield, NHI = nitrogen harvest index, AGDM = above-ground dry matter, PW = pod walls, SL = stems and leaves, TR = total residues. Significance levels are at *p* < 0.05 (*), *p* < 0.01 (**) and *p* < 0.001 (***). There were no significant interactions of S × Yr and V × S × Yr. Different letters indicate significant differences between values.

The NHI was ranked as follows: 2013/14—Diva, Alexia > Hiverna; 2014/15—Diva ≥ Alexia ≥ Hiverna. It was higher for all three varieties in 2014/15 than in 2013/14, with larger differences for Hiverna. The NHI did not differ between seeding rates (Table 1 and Table 4).

### 3.3. NY_GRAIN_ on the Single Plant Level

The mean NY_GRAIN_ on the single plant level over all varieties, seeding rates and years were as follows: NY_GRAIN_ plant^−1^: 407 mg, NY_GRAIN_ stem^−1^: 214 mg, NY_GRAIN_ pod^−1^: 54.7 mg and NY_GRAIN_ grain^−1^: 16.9 mg. 

The NY_GRAIN_ plant^−1^ among varieties was generally ranked as follows: Diva > Hiverna > Alexia. Diva and Hiverna had a higher NY_GRAIN_ plant^−1^ at 25 than at 50 seeds m^−2^, whereas no difference between seeding rates was found for Diva (Table 1 and Table 3). The NY_GRAIN_ plant^−1^ of Diva was also higher in 2014/15 than in 2013/14, whereas there was no difference between years for Hiverna and Alexia (Table 4).

The NY_GRAIN_ stem^−1^ was ranked for seeding rates as follows: 25—Diva > Alexia > Hiverna; 50—Alexia ≥ Diva ≥ Hiverna (Table 3). The NY_GRAIN_ stem^−1^ of Diva was higher at 25 than at 50 seeds m^−2^, whereas it did not differ between seeding rates for Hiverna and Alexia (Table 3). Diva and Hiverna had a higher NY_GRAIN_ stem^−1^ in 2014/15 than in 2013/14, whereas there was no difference between years for Alexia (Table 4).

The NY_GRAIN_ pod^−1^ was ranked as follows: 2013/14—Hiverna, Diva > Alexia; 2014/15—Hiverna > Diva > Alexia; with values of Diva and Hiverna being 1.72 or 1.87-fold higher than those of Alexia (Table 4).

The NY_GRAIN_ grain^−1^ was ranked as follows: Hiverna > Diva > Alexia. The NY_GRAIN_ grain^−1^ of Diva and Hiverna were 1.41-fold or 1.72-fold higher compared to Alexia (over all seeding rates and years).

**Table 2:** Yields on the single plant level, nitrogen utilization, soil mineral nitrogen, nitrogen fixation and nitrogen balance of faba bean as affected by variety, seeding rate and year.

	NY_GRAIN_ (mg)				NUtE (g g^−1^)		SMN	∆ NY_AGDM_	SMN Sparing	N_FIX_	Ndfa	N_FIX-AGDM_	N Balance
	plant^−1^	shoot^−1^	pod^−1^	grain^−1^	AGDM	GRAIN	g m^−2^	g m^−2^			%	mg g^−1^	g m^−2^
**Variety (V)**													
Diva	623 ^a^	256 ^a^	61.5 ^b^	17.3 ^b^	38.2 ^b^	16.5 ^a^	5.29 ^b^	8.29 ^a^	3.55 ^b^	11.83 ^a^	56.6 ^a^	15.0 ^a^	−4.65
Hiverna	356 ^b^	167 ^c^	67.0 ^a^	21.1 ^a^	46.9 ^a^	14.1 ^b^	4.84 ^b^	6.49 ^a^	3.11 ^b^	9.59 ^b^	49.5 ^a^	10.8 ^b^	−2.91
Alexia	243 ^c^	220 ^b^	35.8 ^c^	12.3 ^c^	40.9 ^b^	17.1 ^a^	6.96 ^a^	-0.42 ^b^	5.22 ^a^	4.80 ^c^	34.1 ^b^	9.3 ^b^	−4.73
**Seeding rate (S) (m^−2^)**													
25	523 ^a^	250 ^a^	54.9	16.8	41.7	16.3	5.49	4.46	3.75	8.21	42.0	11.0	−4.57
50	292 ^b^	178 ^b^	54.6	17.0	42.4	15.5	5.90	5.11	4.17	9.27	51.5	12.5	−3.36
**Year (Yr)**													
2013/14	370 ^b^	179 ^b^	53.7	17.8 ^a^	41.9	14.9 ^b^	7.23 ^a^	5.73 ^a^	3.90	9.63	54.4 ^a^	13.2 ^a^	−2.31 ^a^
2014/15	445 ^a^	250 ^a^	55.8	16.1 ^b^	42.1	16.9 ^a^	4.16 ^b^	3.84 ^b^	4.02	7.86	39.1 ^b^	10.3 ^b^	−5.62 ^b^
**ANOVA**													
V	***	***	***	***	***	***	*	***	*	***	**	**	
S	***	***											
Yr	*	***				***	***	*			**	*	***
V × S	***	*										*	
V × Yr	***	**	**	**	**							**	
S × Yr						*							

NY = nitrogen yield, NUtE = nitrogen utilization efficiency, SMN = soil mineral nitrogen, ∆ NY_AGDM_ = difference of the NY in the above-ground dry matter (AGDM) of the faba bean and the reference crop winter wheat, N_FIX_ = nitrogen fixation, Ndfa = nitrogen derived from atmosphere. Significance levels are at *p* < 0.05 (*), *p* < 0.01 (**) and *p* < 0.001 (***). There were no significant interactions of V × S × Yr. Different letters indicate significant differences between values.

**Table 3:** Yield and nitrogen parameters of faba bean as affected by variety × seeding rate.

			Variety			
		Seeds (m^−2^)	Diva	Hiverna	Alexia	LSD
Y_GRAIN_	g m^−2^	25	373	269	179	57
		50	314	269	230	
NY_AGDM_	g m^−2^	25	21.6	18.3	10.5	n. s.
		50	19.6	19.3	13.4	
NY_GRAIN_	g m^−2^	25	17.7	12.6	8.0	2.7
		50	15.3	12.4	10.2	
NY_GRAIN_	mg plant^−1^	25	831	154	283	104
		50	415	258	203	
NY_GRAIN_	mg stem^−1^	25	323	182	244	50
		50	189	152	195	
∆ NY_AGDM_	g m^−2^	25	9.30	5.96	−1.88	n. s.
		50	7.28	7.01	1.04	
N_FIX-AGDM_	mg g^−2^	25	15.5	11.0	6.4	4.2
		50	14.6	10.6	12.3	

Y = yield, NY = nitrogen yield, N_FIX_ = nitrogen fixation, AGDM = above-ground dry matter, LSD = least significant difference, n. s. = not significant.

**Table 4:** Yield and nitrogen parameters of faba bean as affected by variety × year.

			Variety			
		Year	Diva	Hiverna	Alexia	LSD
Y_SL_	g m^−2^	2013/14	449	555	239	48
		2014/15	273	538	209	
NC_GRAIN_	%	2013/14	4.67	4.50	4.51	0.13
		2014/15	4.96	4.77	4.42	
NC_PW_	%	2013/14	1.41	1.37	1.38	0.14
		2014/15	1.66	1.41	1.40	
NC_SL_	%	2013/14	0.95	1.30	1.21	n. s.
		2014/15	0.67	0.73	0.62	
NHI	%	2013/14	73.8	56.6	73.1	6.5
		2014/15	84.6	74.7	79.6	
NY_GRAIN_	mg plant^−1^	2013/14	493	374	242	104
		2014/15	753	338	245	
NY_GRAIN_	mg stem^−1^	2013/14	188	139	209	50
		2014/15	324	195	230	
NY_GRAIN_	mg pod^−1^	2013/14	60.2	61.9	38.9	6.4
		2014/15	62.8	72.2	32.6	
NY_GRAIN_	mg grain^−1^	2013/14	18.9	20.9	13.6	1.7
		2014/15	15.8	21.4	11.0	
NUtE_AGDM_	g g^−1^	2013/14	41.3	46.5	38.0	4.6
		2014/15	35.2	47.3	43.9	
N_FIX-AGDM_	mg g^−2^	2013/14	14.0	11.7	13.9	4.2
		2014/15	16.0	9.9	4.8	

Y = yield, NC = nitrogen concentration, NHI = nitrogen harvest index, NY = nitrogen yield, NUtE = nitrogen utilization efficiency, AGDM = above-ground dry matter, SL = stems and leaves, PW = pod walls, LSD = least significant difference, n. s. = not significant.

### 3.4. Nitrogen Utilization

The mean NUtE over all varieties, seeding rates and years was for NUtE_AGDM_: 42.0 g g^−1^ and for NUtE_GRAIN_: 15.9 g g^−1^. 

The NUtE_AGDM_ among varieties was ranked as follows: 2013/14—Hiverna > Diva ≥ Alexia; 2014/15—Hiverna, Alexia > Diva. Diva had a higher NUtE_AGDM_ in 2013/14 and Alexia in 2014/15 when compared to the other year, while no difference between years was observed for Hiverna (Table 4). No difference was observed between the seeding rates (Table 2).

The NUtE_GRAIN_ was ranked among varieties as follows: Hiverna > Alexia and Diva (Table 2). The NUtE_GRAIN_ of was higher in 2013/14 at 25 than at 50 seeds m^−2^, whereas no differences were observed between the seeding rates in 2014/15 (in g g^−1^; 25 versus 50 seeds m^−2^: 2013/14—15.7 or 14.1; 2014/15—16.9 or 17.0; LSD = 1.1).

### 3.5. Soil Mineral Nitrogen, Nitrogen Fixation and Nitrogen Balance at Harvest

The mean values over all varieties, seeding rates and years for SMN at harvest in a soil depth of 0–0.9 m were 5.70 g m^−2^, ∆ NY_AGDM_: 4.78 g m^−2^, SMN sparing: 3.96 g m^−2^, N_FIX_: 8.74 g m^−2^, Ndfa: 46.7%, N_FIX-AGDM_: 11.7 mg g^−1^ and N balance: −3.96 g m^−2^.

The SMN was ranked as follows: variety—Alexia > Diva, Hiverna; year—2013/14 > 2014/15; seeding rate did not affect SMN (Table 2). 

The ∆ NY_AGDM_ was ranked as follows: variety—Diva, Hiverna > Alexia; year—2014/15 > 2013/14. The ∆ NY_AGDM_ of Alexia tended to be higher at 50 compared to 25 seeds m^−2^, with no difference for Diva and Hiverna (*p* = 0.066, n. s.) (Table 2 and Table 3).

The SMN sparing ranked among varieties as follows: Alexia > Diva, Hiverna. For Alexia it was 1.47-fold or 1.68-fold higher compared to Diva and Hiverna. It did not differ between seeding rates and years (Table 2). 

The N_FIX_ was ranked among varieties as follows: Diva, Hiverna > Alexia. For Diva and Hiverna, it was 2.47-fold or 2.00-fold higher compared to Alexia. The N_FIX_ did not differ between seeding rates. It tended to be higher in 2013/14 than in 2014/15 (*p* = 0.054, n. s.) (Table 2).

The Ndfa ranked among varieties as follows: Diva, Hiverna > Alexia. For Diva and Hiverna, it was 1.65-fold or 1.45-fold higher compared to Alexia. The Ndfa tended to be higher at 50 compared to 25 seeds m^−2^ (*p* = 0.064, n. s.). It was 1.39-fold higher in 2013/14 than in 2014/15 (Table 2).

The N_FIX-AGDM_ among varieties at different seeding rates was as follows: 25—Diva, Hiverna > Alexia; 50—Hiverna ≥ Diva ≥ Alexia. It did not differ between years (Table 2 and Table 3).

The N balance was negative for both years, with a higher negative value in 2014/15 than in 2013/14. It did not differ between seeding rates. It tended to be more strongly negative for Diva than for Hivena (*p* = 0.096, n. s.) (Table 2).

## 4. Discussion

Winter faba beans had generally higher NY_GRAIN_ and NY_TR_ than the spring faba bean. Details are shown in Neugschwandtner et al. [11]. A higher Y_GRAIN_ results from the higher crop plasticity in the formation of yield components, which becomes more evident with a longer growing period and with earlier seeding [32]; this can also be a result of relatively higher resistance to anthracnose of winter faba varieties compared to the spring varieties, which can cause high yield losses [33]. Nevertheless, winter hardness is important for a good yield of winter faba bean [34]. Low winter survival results in no yield advantage of winter over spring faba bean [35].

NC_GRAIN_ and NC_PW_, but not NC_SL_, were higher in winter faba beans than in the spring faba bean. Seeding rate did not affect NC in plant, although a higher protein concentration was observed at a higher seeding rate for soybean [16,21].

Just as both Y_GRAIN_ and NC_GRAIN_ were higher in winter faba beans compared to the spring faba bean, the NY_GRAIN_ was also considerably higher. The highest NY_GRAIN_ was obtained with 16.5 g m^−2^ by Diva, thereby having a 1.80-fold higher value than Alexia. Under much colder and wetter conditions in south-western Germany, a NY_GRAIN_ of 22 g m^−2^ was achieved with spring faba bean [36]. Faba bean is widely used for feed and food due to its good nutritional value. One aspect here is the protein content [1]; regarding this aspect, Diva and Hiverna are more favourable than Alexia.

Considerably higher Y_TR_ of winter faba beans, despite lower NC_PW_, resulted in higher NY_TR_ compared to the spring faba bean. Similar to our observations, Aufhammer et al. [36] reported a positive correlation of Y_GRAIN_ and NY_TR_, concluding that conditions which enable the formation of a high Y_GRAIN_ also contribute significantly to a higher NY_TR_. Based on results of our study, this optimum condition would be seeding winter faba bean and not spring faba bean. The highest NY_TR_ was observed for Hiverna, with 6.33 g m^−2^, which was below values reported for spring faba bean grown in south-western Germany, with values ranging from 7 to 17 g m^−2^ [37], though shed leaf litter was not sampled in our study. The share of shed leaf litter as part of the AGDM of spring faba bean was reported at 6% [38] or 25% [36]. Considering a this additionally, the NY_TR_ and thereby resulting positive pre-crop effects might be even higher. Higher NC_GRAIN_ but lower NC_PW_ resulted in a higher NHI compared to the harvest index (HI) for winter faba bean, whereas the HI and the NHI for the spring faba bean was in a similar range (cf. HI values [11]).

A yield component analysis for this experiment (cf. [11]) showed that Y_GRAIN_ plant^−1^ was ranked as follows: Diva > Hiverna > Alexia. Considering additionally the higher NC_GRAIN_ of winter faba bean, the NY_GRAIN_ plant^−1^ was higher in winter faba beans than in the spring faba bean. Hiverna had the highest number of stems plant^−1^; consequently, the NY_GRAIN_ shoot^−1^ was lower than that of Diva and Alexia. The higher Y_GRAIN_ pod^−1^ of winter faba beans resulted from the higher TKW and a higher number of grains pod^−1^ (except for Hiverna in one year). Considering additionally the higher NC_GRAIN_ of winter faba beans, the NY_GRAIN_ pod^−1^ of winter faba beans was almost two times higher than that of Alexia. The considerably higher NY_GRAIN_ grain^−1^ of Diva and Hiverna compared to Alexia resulted from both the higher NC_GRAIN_ and the higher TKW.

The NUtE gives the yield per unit N in the above-ground dry matter; thus, higher values show that more biomass could be produced with the N which was taken up. Hiverna had the highest NUtE_AGDM_ among varieties but, due to the high residue yields and the low NHI, the lowest NUtE_GRAIN_, whereas those of Diva and Alexia did not differ. However, autumn-sowing of facultative wheat resulted in a higher NUtE_AGDM_ than spring-sowing [5]. The NUtE_GRAIN_ was higher in the drier vegetation period 2014/15. Contrary to that, the NUtE_GRAIN_ of barley, oat and pea were severely decreased by drought [39]. In one year, the NUtE_GRAIN_ was higher for all varieties at the lower seeding rate. Interference during grain formation may cause a lower NUtE, as reported in triticale–faba bean intercrops [40] and oat–pea intercrops [41]. Further, N fertilization decreases the NUtE [42,43]. The NUtE differed between winter faba bean varieties, as Hiverna had a lower NHI than Diva. Contrary to that, no differences in the NUtE were reported for spring barley varieties [44].

SMN and SMN sparing were higher in Alexia than in Diva and Hiverna. A higher residual nitrate content in the soil at harvest of faba bean compared to oat was attributed to lower rooting density and, thereby, the lower nitrate uptake of faba bean [44]. Assuming that the spring faba bean had not only a lower Y_AGDM_ compared to winter faba bean but also a lower rooting density, this might be the reason for both the higher SMN and SMN sparing.

The mean value of N_FIX_ of Diva and Hiverna were with 11.83 or 9.59 g m^−2^ considerably higher than that of Alexia (4.80 g m^−2^). At the same location, values for N_FIX_ for winter faba bean of 6.3 g m^−2^ due to low overwintering and of 21.9 g m^−2^ under optimum conditions have been reported [45]. Values of N_FIX_ for spring faba bean and spring pea in Bavaria (Germany) ranged between 16.5 and 24.0 g m^−2^ for spring faba bean and between 21.5 and 24.6 g m^−2^ for spring pea [46].

The higher N_FIX_ of Diva and Hiverna resulted mainly from their higher ∆ NY_AGDM_, which for each variety was more than three times higher than their SMN sparing. The positive value of N_FIX_ of Alexia, however, resulted solely from SMN sparing, as its ∆ NY_AGDM_ was negative, with a lower NY_AGDM_ than the reference crop winter wheat. 

Positive yield effects of legumes on subsequent crops have been attributed to the release of biologically-fixed N from residues and the SMN sparing of legumes [38,47]. Consequently, positive pre-crop effects of winter faba bean might better attributed to the release of biologically-fixed N from residues compared to the SMN sparing; however, it is the other way around for spring faba bean.

In a climate change simulation study for conditions in south-western France, the simulated N_FIX_ of winter faba bean and winter pea were enhanced by the increased temperature during their vegetative growth in winter, whereas the N_FIX_ of spring pea was reduced by an above-optimal temperature during its vegetative growth in spring [2]. The lower N_FIX_ in 2014/15 compared to 2013/14 might be explained by less rainfall during the vegetation period, as water stress is the overriding factor for driving yield and N_FIX_ of faba bean [2].

The highest value for Ndfa was seen in Diva, at 56.6%, and the lowest in Alexia, at 34.1%. Zapata et al. [48] even reported a value of 79% for Ndfa for faba bean. The percentage of Ndfa for winter faba beans was considerably higher than for the spring faba bean. Also, autumn-sown chickpea was reported to have a higher Ndfa than spring-sown chickpea [17]. 

The mean N_FIX-AGDM_ was 11.7 mg g^−1^, with higher values for Diva than for Alexia. The N_FIX-AGDM_ for spring pea grown at the same location in pure stands and intercrops with oat was 22.9 mg g^−1^, as the N_FIX_ was with 18.51 g m^−2^, also more than two times higher than the mean value in our study [49]. Also, a higher N_FIX_ for winter pea than for winter faba bean under Pannonian climate conditions has already been reported [10].

The N balance for all varieties had negative values, as N_FIX_ was lower than NY_GRAIN_. However, the negative N balance of Diva and Alexia was only half that of the reference crop winter wheat. Also, the N balance of spring faba bean grown in northern Germany was negative at −5.7 g m^−2^, whereas that of spring pea was positive at 1.9 g g m^−2^ [31]. Senaratne and Hardarson [50] also observed a negative N balance for faba bean, reporting that it had depleted the soil more than fallow but less than barley.

Hiverna had the lowest negative N balance (n. s.) due to having the lowest NHI. The negative N balance increases with a higher harvest index and a higher N harvest index, as more N is transferred from the field [31]. The N balance was more than twice as negative in 2014/15 than in 2013/14, as in 2014/15 the NY_GRAIN_ tended to be higher (n. s.) while N_FIX_ was lower.

## 5. Conclusions

Winter faba bean is superior to spring faba bean in achieving high NY and high N_FIX_, due to not only higher biomass yields, but also a higher NC_GRAIN_ and a higher Ndfa. The positive pre-crop effect of winter faba bean might be higher because more N is left in the field, mainly in the residues, though spring faba bean has a higher SMN sparing. Winter faba bean can be sown with a low seeding rate of 25 germinable seeds m^−2^, whereas the seeding rate of 50 germinable seeds m^−2^ for the spring faba bean results in a higher NY_GRAIN_. Among winter faba bean varieties, Diva had a higher Y_GRAIN_, NC_GRAIN_, NY_GRAIN_, NHI and N_FIX_, whereas Hiverna had a higher Y_SL_, NY_TR_ and a less negative N balance.

## Data Availability

The data presented in this study are available on request from the corresponding author.
